# Construction of a Matrix

**Published:** 1903-05-15

**Authors:** G. W. Dittmarr


### CONSTRUCTION OF A MATRIX.

The material used may be either copper, German silver or steel, rolled to from 32 to 35 B. & S. gauge. From this cut a piece long enough that, when adjusted to an ordinary proximal complex cavity, for example, a disto-occlusal in a bicuspid or molar, the ends will extend a few millimeters beyond the cavity margins into the buccal and lingual embrasures, and wide enough to extend from the occlusal to a trifle beyond the gingival wall. Then with curved crown shears and contouring pliers, cut and contour this band of metal to properly fit the case at hand. Then roughen the ends, which are to lie against the buccal and lingual surfaces of the tooth, by simply cutting five or six slits in each, not more than one-half millimeter in depth. A small hole made with a plate punch near the buccal and lingual corners next the gingival edge (through which the ligature is to be passed before adjusting the matrix), will prove a great convenience.

If the matrix is long—that is, if it is necessary to restore lost buccal and lingual walls, it is advisable to cut a couple of slits about one millimeter in depth, and a like distance apart, on the gingival edge of the band; then turn the flap between the two slits toward the occlusal thus forming a little hook, which should of course, be externally. Make one of these hooks near the buccal and lingual embrasures on the gingival edge, and if thought necessary, on the occlusal edge also, the ends of the occlusal hooks to be turned gingivally.

Having thus, in a few minutes, constructed the matrix desired, place it, and hold it in position by tying with a waxed silk ligature, encircling the tooth, not less than three, and possibly five or six times. The roughened ends and little holes or hooks will greatly assist in retaining the ligature in its proper position.—G. W. Dittmarr, in *Review*.

